# When Should the Electrophysiologist Be Involved in Managing Patients with Ventricular Assist Devices and Ventricular Arrhythmias?

**DOI:** 10.19102/icrm.2019.100407

**Published:** 2019-04-15

**Authors:** Jeffrey S. Arkles, Francis Marchlinski

**Affiliations:** ^1^Electrophysiology Section, Division of Cardiovascular Medicine, University of Pennsylvania, Philadelphia, PA, USA

**Keywords:** ICD, LVAD, surgical ablation, ventricular arrhythmia

## Abstract

The successful management of ventricular arrhythmias (VAs) in people with left ventricular assist devices (LVADs) is often complex. The need for and the role of defibrillator therapy is continually evolving in this group. VAs occur frequently and significantly impact the clinical course of patients with LVADs. The management of VAs begins prior to LVAD implantation and typically involves appropriate implantable cardioverter-defibrillator use and programming after the fact. Surgical ablation during LVAD implantation and supplementary catheter ablation performed as needed are attractive options for the management of VAs in this population. The performance of catheter ablation is generally safe and feasible after LVAD implantation with a team approach.

## Introduction

Continuous-flow left ventricular (LV) assist devices (LVADs) have revolutionized the care of patients with severe heart failure. Their use is expanding, and LVAD destination therapy may represent an alternative to transplantation in some younger patients. More than 22,000 patients have received mechanical circulatory support as of the beginning of 2017.^1,2^ Ventricular arrhythmias (VAs) are a common comorbidity of LVAD use. The burden of VAs in LVAD patients can reach more than 40% within two years, with a resulting significant impact on the quality of life.^3^

The management of VAs in the LVAD population requires a nuanced approach. A not uncommon clinical scenario is that a patient with an LVAD presents with sustained ventricular tachycardia (VT) or ventricular fibrillation (VF) with minimal symptoms.^2^ In particular, patients with an LVAD and VAs in the setting of right ventricular (RV) dysfunction and high pulmonary vascular resistance (PVR) are at risk for severe RV deterioration. A high VA burden can induce negative remodeling and further worsen cardiac function.^4^

Patients with VAs before LVAD implantation have a higher rate of VAs after device placement; however, patients with no such history are still at risk for new VAs following LVAD implant as well. Other predictors of VAs arising after LVAD implantation include a history of atrial fibrillation. Conversely, type of cardiomyopathy, history of valve disease, and LV dimension do not confer a significant risk.^5^

The role of implantable cardioverter-defibrillators (ICDs) is evolving and becoming highly individualized in patients with LVADs. Recent studies have failed to show a mortality benefit with ICD use in LVAD patients.^6,7^ However, in some LVAD patients, VAs can be life-threatening and associated with hemodynamic collapse, and ICD shocks in these individuals can be life-saving.

Similarly, the management of VAs is highly individualized as well. When the LVAD is destination therapy and transplant is not an option, the management of VAs can be more urgent. Separately, when transplant is an option, aggressive alternatives may not be relevant. VAs that are well-tolerated and self-terminating may not necessitate aggressive treatment. In patients with RV dysfunction or pulmonary vascular disease, the impact of VAs is often much greater and warrants more aggressive treatment.

## When should the electrophysiologist be involved?

### Assessment of ventricular arrhythmias before left ventricular assist device implantation and the planning of surgical ablation

**Figure 1:**
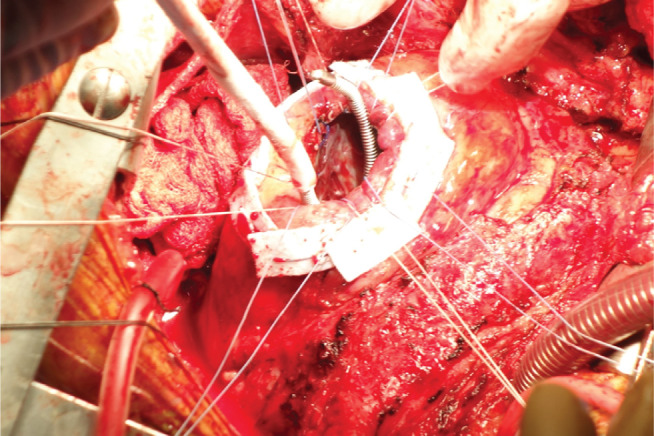
Surgical cryoablation performed at the time of LVAD implantation. The apex is exposed and the cryocatheter is applied endocardially to arrhythmogenic sites.

The most significant electrophysiology consultation generally occurs before LVAD implantation. Patients with VAs prior to LVAD placement are at the highest risk for recurrent arrhythmias post-LVAD insertion, with a 19-fold increase in the risk for post-LVAD VAs.^3^ The LVAD implantation procedure offers a unique opportunity for surgical VT ablation to be performed during cardiopulmonary bypass. Notably, the efficacy of cryoablation in a cardioplegic heart is far greater than that of conventional ablation^8^ **(Figure 1)**. This fact furthermore highlights the importance of employing a team approach in these cases, with members offering heart failure, electrophysiology, and surgical expertise.^9,10^

At our institution, in patients with VAs who are considering undergoing LVAD implantation, we attempt to identify the substrate generating the VA. This may be done through invasive electroanatomic mapping, imaging, electrocardiography, or a combination of these three techniques. In some cases, there is an obvious substrate such as an apical aneurysm that correlates with an apical origin of VT. Conversely, in other instances where the scar is more diffuse, electroanatomic mapping and/or imaging can provide additional details about the origin of the arrhythmogenic substrate.

Based on the anatomical location of this substrate, catheter ablation can be performed or deferred if the patient is hemodynamically unstable. VTs that may arise from the basal septal segments of the heart tend to be more challenging to visualize and access surgically. Attempts at catheter ablation prior to LVAD placement are worthwhile in this case.^11^ In patients with large scar burdens such as those with anterior myocardial infarction, we often recommend prophylactic surgical ablation in patients without a history of VAs.

### Electrophysiology consultation for considering implantable cardioverter-defibrillator placement and sudden cardiac death risk following ventricular assist device implantation

The consideration of ICD post-LVAD insertion may be worthwhile. The literature has evolved over the recent years and now indicates the existence of a questionable benefit with ICD therapy. Some degree of increased risk has been previously described.^12,13^ Since there is currently no randomized trial either ongoing or complete that is designed to answer this question, the decision to implant an ICD must be made on an individual basis. We generally recommend ICD placement for secondary prevention in the LVAD population. The presence of early postoperative VAs, especially when the patient is on high-dose pressor support, may not warrant ICD therapy.

Patients with abnormal RV function or elevated PVR are most at risk for hemodynamic collapse related to sustained VAs. However, these are individuals in whom lead-related tricuspid regurgitation has the potential for greatest harm. While a subcutaneous ICD is an option in these patients, there have been issues with sensing and inappropriate therapy reported.^14,15^ Therefore, ICD use in this population cannot be recommended without carefully weighing the risks and benefits.

Separately, in patients with no history of VAs, we do not recommend routine ICD implantation. Our clinical experience has matched findings in other published reports suggesting that these patients in general do well from an arrhythmia perspective.^13^

### Electrophysiology consultation after left ventricular assist device placement in patient with existing implantable cardioverter-defibrillator

After LVAD implantation, VAs are common and, thus, established preimplant ICD detection criteria may not always be appropriate. Our practice is to increase detection times for VT to near the maximum, depending on the manufacturer. VF zones are generally increased to 10 seconds or more. Often, shocks are turned off in VT zones and are left on in VF zones. However, variations of this conservative programming strategy have also failed to reduce ICD shocks in this population.^16^

ICD system damage can occur following LVAD implantation either due to lead fracture or a change in sensing related to the apical surgery.^17^ A drop in RV sensing amplitude often occurs. If the amplitude drops to a level at which VA detection is questionable and there is a history of VA, we recommend defibrillation threshold testing. Lead revision is avoided whenever possible due to the perioperative risks of bleeding and infection and the enhanced risk of tricuspid regurgitation and RV dysfunction with implantation of a new lead or lead extraction. There are some data that suggest these changes in lead parameters are transient phenomena.^18^

The role of cardiac resynchronization therapy (CRT) in patients with LVADs is unclear. The LV is unloaded from a hemodynamic perspective and CRT is unlikely to add additional cardiac output. However, a positive response to CRT may be associated with fewer arrhythmias, likely due to positive remodeling and neurohormonal changes in the LV.^19^ Some studies have suggested that interrupting CRT in LVAD patients may be associated with worsened VA outcomes.^20^ The need for more frequent generator changes is also a legitimate concern. Our general practice is to maintain CRT, although there are some cases where high thresholds and rapid battery depletion would lead us to disable it for the sake of avoiding a generator change.

### Electrophysiology consultation for ventricular arrhythmias after left ventricular assist device implantation

#### Ruling out mechanical causes

**Figure 2:**
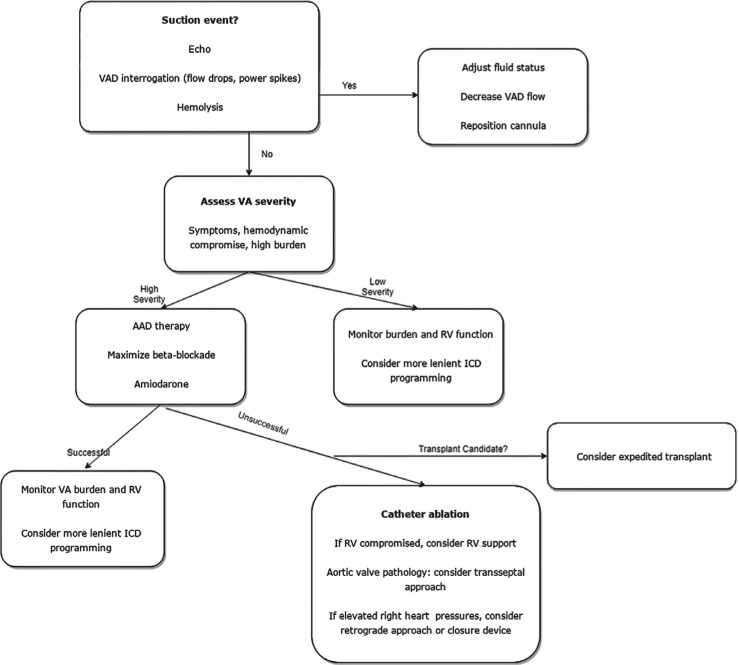
Evaluating VAs in patients with LVADs. AAD: antiarrhythmic drug; ICD: implantable cardioverter-defibrillator; RV: right ventricle/ventricular; VA: ventricular arrhythmia; VAD: ventricular assist device.

Our overall strategy is summarized in **Figure 2**. As mentioned previously, mechanical causes of VA, such as the cannula contacting the myocardium, may occur. These mechanical interactions often transpire in smaller hearts or with more septal cannula orientation. Fluid and/or RV status play a role in the relative underfilling of the LV. Often, these suction events can be diagnosed by characteristic dips in LVAD flow tracings, via echocardiography findings, and with evidence of hemolysis. Rarely, the cannula position can be persistently problematic and require surgical revision.

#### Immediate postoperative ventricular arrhythmias

Immediate postoperative VAs are common and can be self-limited.^5^ Often, high-dose inotropic and pressor support after surgery can exacerbate arrhythmias. However, unremitting VT storm post-LVAD can occur even in patients with no history of VAs. Our approach is generally to treat with antiarrhythmic drugs (AADs). Amiodarone is typically chosen as the drug of choice through this period, with lidocaine constituting an alternate therapy. Weaning of inotropes is critical and, if possible, β-blockade and sedation can be useful.

If reasonable VA suppression can be achieved, patients can be weaned from amiodarone over time in the outpatient setting. Prior research has shown that, despite treatment with amiodarone and other AAD therapy, VAs are often recurrent.^21^ If there is not a good response to medical therapy, catheter ablation during this early postoperative time period can also often be performed successfully.

#### Ventricular arrhythmias after the early postoperative period

After suction-cannula-related events have been ruled out, the nature and scope of VAs are determined. If the VAs are self-terminating and the patient is asymptomatic with good RV function, there is little need for aggressive medical or ablative treatment. Heart failure is aggressively treated, the fluid status is optimized, and the patient is further monitored.

However, if there are significant symptoms including syncope, dizziness, or fatigue associated with VAs, more aggressive treatment is warranted. Additionally, if a patient is felt to be particularly vulnerable due to tenuous RV function, treatment is offered. Typically, amiodarone is the drug of choice. Sotalol is reasonable in some patients, but the β-blocker effect on RV function must be monitored. Mexiletine can play an adjunctive role with amiodarone. Amiodarone should be used with caution, however, because of the potential for interactions with warfarin and hepatic side effects. If these medical therapies are ineffective, catheter ablation is the preferred therapy.

### Catheter ablation in patients with left ventricular assist devices

VAs are often not adequately suppressed with AAD therapy. Catheter ablation is thus an attractive alternative option in these patients or in those experiencing side effects from AAD therapy. As mentioned previously, in asymptomatic patients with normal RV function, a greater burden of VA can be tolerated. However, prolonged VT even in patients without gross RV dysfunction can contribute to hemodynamic collapse. It is also important to consider the primary goal of the LVAD implant; specifically, if the patient is receiving destination therapy, catheter ablation may be the only option. If transplant is an option, efforts to expedite transplant status are prudent.

**Figure 3:**
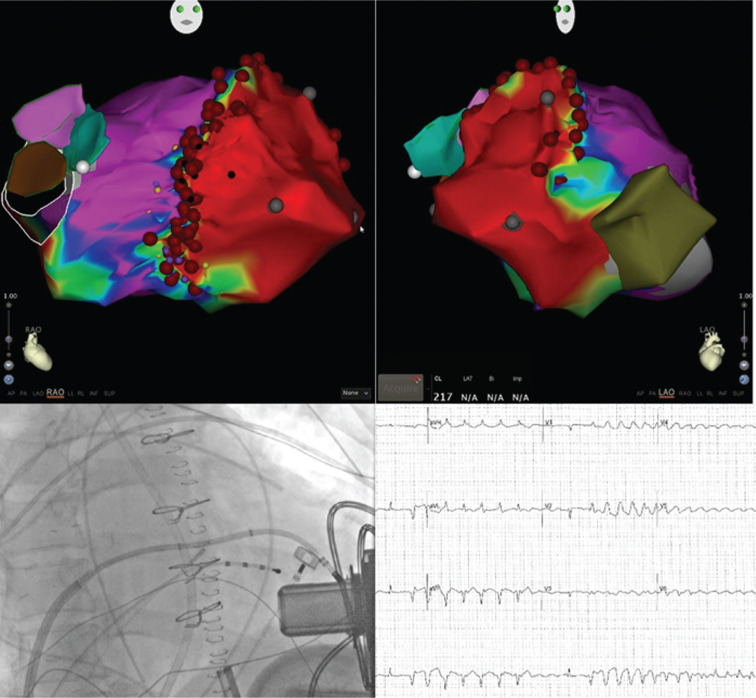
Patient with a large anterior infarct requiring LVAD implant and no history of VT. He developed incessant VT after LVAD implantation that was refractory to AAD. **A and B:** Three-dimensional electroanatomic maps of the HeartWare LVAD (HeartWare International Inc., Framingham, MA, USA) and large anteroapical scar. **C:** Transseptal approach to catheter ablation with the catheter balanced on the inflow cannula of the VAD. **D:** Clinical arrhythmia (apical superiorly directed VT) originating from near the LVAD cannula.

Catheter ablation of VT in LVAD patients has been proven to be safe and effective. The majority of the arrhythmogenic substrate is not directly related to the inflow cannula but rather to the preexisting substrate.^22^ This highlights the importance of electrophysiology consultation prior to LVAD implantation. In cases of VAs after implantation, VT ablation can be performed safely **(Figure 3)**.

An important consideration with catheter ablation is the status of the aorta and aortic valve. In some cases where aortic regurgitation (AR) is significant, the aortic valve may be oversewn at the time of VAD placement. In other cases, there may be minimal pulsatility of the LV and minimal aortic valve opening. Often, turning down the flow rate temporarily can enhance the aortic valve opening and therefore more easily allow for catheter passage. Some operators prefer to cross with a straight, floppy-tipped wire and then exchange this for a long sheath such as the SL1 Fast-Cath (Abbott Laboratories, Chicago, IL, USA). The consideration of worsening AR is important especially where there is significant preexisting AR, as this can dramatically worsen VAD hemodynamics. In cases where there is significant AR or arterial pathology, we would consider a transseptal approach.

When a transseptal approach is under consideration, RV function and pulmonary hypertension are of critical importance. When right atrial pressure is more than left atrial pressure, the risks of shunting and hypoxia as well as paradoxical emboli are significantly increased. Pulmonary artery catheterization is typically performed before transseptal access. If the right atrial pressure is significantly more than left atrial pressure, either a retrograde approach is used or a closure device is considered following the ablation procedure.

RV function and PVR are also important considerations to keep in mind during catheter ablation in general. The LVAD can generally support prolonged mapping in VT so long as there is enough preload. The conditions for preload to be adequate include a favorable PVR and adequate volume status. Inhaled epoprostenol can be used to help lower the PVR during these cases; however, even then, prolonged VT in a vulnerable RV can lead to hemodynamic deterioration.

Direct RV support in the form of bypass or peripheral VAD placement (Impella RP; Abiomed, Danvers, MA, USA) can also be used. This allows for safer mapping of VT without concern for acute RV failure. These interventions require close collaboration of the perfusion, surgery, and anesthesiology teams.

Ablation is directed toward the clinical VT. Often, there are large areas of low voltage that can be difficult to fully homogenize. The hemodynamic support of the LVAD allows for extensive mapping of the tachycardia. A VAD-trained nurse or physician needs to be present during the entirety of the case. In instances where the RV is not supported, acute drops in VAD flow may be noted in VT.

Percutaneous epicardial access is not generally attempted. Additional substrate-based ablation targeting late and fragmented potentials is typically performed. The HeartWare VAD (HeartWare International Inc., Framingham, MA, USA) produces significant magnetic interference with magnetically tracked catheters when in close proximity to the outflow region. Generally, the arrhythmogenic substrate is not in close proximity to the cannula, and this is not a significant clinical issue.^22^ Intracardiac echocardiography can be useful in identifying intracavitary structures, scar, and complications. It is particularly useful in assessing LV filling during VT.

In general, the end goal is noninducibility of clinical VT. Given the substantial multidisciplinary effort required to bring these patients to the electrophysiology laboratory, it makes sense to target all instances of inducible VT by way of substrate-based ablation as well. This must be balanced against the comorbidities of the patient and higher rates of stroke and bleeding in this population. Alternative options for management include stellate ganglion block for incessant VAs, which has shown some degree of success.^23^ However, in our own clinical experience, favorable outcomes have been limited with this therapy.

## Conclusions

Electrophysiologists should work in close collaboration with cardiac surgery and advanced heart failure physicians before, during, and after LVAD implantation procedures. VAs are common in LVAD patients and can have a significant impact on clinical outcomes. Often, VAs can be addressed successfully during LVAD implantation by way of surgical cryo-based ablation. VAs arising after LVAD placement may be limited and not require aggressive treatment. When clinical circumstances require intervention with VAs, medical treatment with amiodarone or catheter ablation therapy may be appropriate choices. Understanding the best access approaches, appropriate ablation targets, and techniques for overcoming mapping and ablation limitations can produce successful outcomes.
